# Identification of potential susceptibility genes in patients with primary Sjögren’s syndrome-associated pulmonary arterial hypertension through whole exome sequencing

**DOI:** 10.1186/s13075-023-03171-y

**Published:** 2023-09-20

**Authors:** Mucong Li, Yue Shi, Jiuliang Zhao, Qian Wang, Mengtao Li, Xiuli Zhao

**Affiliations:** 1 Department of Medical Genetics, Institute of Basic Medical Sciences & Chinese Academy of Medical Sciences and School of Basic Medicine, Peking Union Medical College, Beijing, China; 2 Department of Rheumatology, Peking Union Medical College Hospital (PUMCH), Peking Union Medical College and Chinese Academy of Medical Sciences, National Clinical Research Center for Dermatologic and Immunologic Diseases (NCRC-DID), Key Laboratory of Rheumatology and Clinical Immunology, Ministry of Education, Chinese Rheumatism Data Center (CRDC), Chinese SLE Treatment and Research Group (CSTAR), State Key Laboratory of Complex Severe and Rare Diseases, Peking Union Medical College Hospital, Chinese Academy of Medical Science and Peking Union Medical College, Beijing, China

**Keywords:** Primary Sjögren’s syndrome, Pulmonary arterial hypertension, Whole-exome sequencing, *FLG*, *BCR*, *GIGYF2*, *ITK*, *SLC26A4*

## Abstract

**Background:**

Pulmonary arterial hypertension (PAH) is a rare complication of primary Sjögren’s syndrome (pSS). Several genes have proven to be associated with pSS and PAH. However, there is no study specifically addressing the genetic susceptibility in pSS combined with PAH.

**Methods:**

Thirty-four unrelated patients with pSS-PAH were recruited from April 2019 to July 2021 at Peking Union Medical College Hospital. Demographic and clinical data were recorded in detail, and peripheral blood samples were collected for whole-exome sequencing (WES). Gene ontology (GO) and Kyoto Encyclopedia of Genes and Genomes (KEGG) pathway analysis were performed to predict the functional effect of mutant genes. Genetic variants identified by WES were confirmed by polymerase chain reaction (PCR)-Sanger sequencing.

**Results:**

We totally identified 141 pathogenic variant loci of 129 genes in these 34 pSS-PAH patients, using WES analysis. Patients with a family history of rheumatic diseases are more likely to carry *FLG* mutations or carry gene variations related to the biosynthesis of the amino acids pathway (*p* < 0.05). According to Sanger sequencing confirmation and pathogenicity validation, we totally identified five candidate pathogenic variants including *FLG* c.12064A > T, *BCR* c.3275_3278dupCCGG, *GIGYF2* c.3463C > A, *ITK* c.1741C > T, and *SLC26A4* c.919-2A > G.

**Conclusion:**

Our findings provide preliminary data of exome sequencing to identify susceptibility loci for pSS-PAH and enriched our understanding of the genetic etiology for pSS-PAH. The candidate pathogenic genes may be the potential genetic markers for early warning of this disease.

**Supplementary Information:**

The online version contains supplementary material available at 10.1186/s13075-023-03171-y.

## Introduction

Primary Sjögren’s syndrome (pSS) is an autoimmune connective tissue disease (CTD) characterized by exocrine gland dysfunction, resulting predominately in dryness of the mouth and eyes [1]. Pulmonary arterial hypertension (PAH) is a major cause of death in CTD patients, with a 5-year survival of 62.9% in China [2]. CTD-associated PAH (CTD-PAH) is classified as group I pulmonary hypertension, which also includes idiopathic PAH (IPAH), heritable PAH (HPAH), PAH due to drugs or toxins, PAH associated with human immunodeficiency virus infection, portal hypertension, congenital heart diseases and schistosomiasis [3]. The most common underlying diseases in Chinese patients with CTD-PAH were systemic lupus erythematosus (SLE), systemic sclerosis (SSc), and pSS [2]. PAH is a rare and severe complication of pSS with poor prognosis [4] and the pathogenesis of pSS-associated PAH (pSS-PAH) is unclear yet.

Mutations in the gene bone morphogenic protein receptor type 2 (*BMPR2*) were reported as the most common genetic cause of PAH and have proven to be associated with long-term outcomes in IPAH, HPAH, and anorexigen-associated PAH [5]. More recently, more IPAH susceptibility genes, including the gene encoding human bone morphogenetic protein 9 (BMP9) and prostacyclin synthase (PTGIS), were identified by employing whole exome sequencing (WES) and functional assessments [6, 7]. Several studies using targeted gene sequencing panels were also conducted in CTD-PAH patients [8, 9]. In addition, genetic studies in pSS have identified mutations in *HLA*, *IRF5*, *STAT4*, *GTF2I*, and *CCL11* (eotaxin) [10–12] genes. However, the susceptibility locus for pSS-PAH remains unknown.

The aim of this study was to explore the genetic susceptibility of pSS-PAH and to establish a preliminary understanding on the association between genotypes and clinical phenotypes.

## Methods

### Study population

A total of 34 pSS-PAH patients were recruited based on a clinical registry in Peking Union Medical College Hospital (PUMCH) between April 2019 and July 2021, a national referral center for CTD-PAH patients. All subjects satisfied the 2002 American–European Consensus Group classification criteria [13] and the 2016 American College of Rheumatology (ACR)/European League Against Rheumatism (EULAR) classification criteria for pSS [14]. Diagnoses of PAH were based on right heart catheterization (RHC), defined as a mean pulmonary arterial pressure (mPAP) ≥ 25 mmHg at rest, a pulmonary artery wedge pressure (PAWP) ≤ 15 mmHg, and a pulmonary vascular resistance of ≥ 3 Wood units (WU) [15]. The exclusion criteria included the presence of any other CTD, left heart disease, interstitial lung disease, and chronic thromboembolic disease confirmed by ventilation perfusion scintigraphy (V/Q) or computed tomographic pulmonary angiography (CTPA). Written informed consent was obtained from all subjects. This study was approved by the Institutional Review Board of PUMCH (JS-2038).

### Data and sample collection

The demographic characteristics, medical history, physical examination findings, laboratory profiles, echocardiography results, RHC data, and treatment information were recorded. The evaluation of pSS was achieved through pSS disease damage index (SSDDI) [16]. Peripheral blood samples were collected from all subjects. DNA was extracted from the peripheral blood by standard procedure based on sodium dodecyl sulfate-proteinase K-phenol/chloroform extraction [17].

### DNA sequencing

Genomic DNA from 34 patients underwent WES. Purified DNA was fragmented, end-repaired, A-tailed, and underwent adaptors ligation and DNA fragments enrichment. Next-generation sequencing was carried out on HiSeq 4000 System (Illumina). Sequencing analysis was performed in all patients using an in-house developed analytical pipeline [18]. The sequencing reads were mapped to the GRCh37/hg19 human reference sequence using the Burrows-Wheeler Aligner (BWA)-MEM alignment algorithm. The BAM files were manipulated by Picard. HaplotypeCaller was used to call potential variant sites. The annotation and filtration of gene variants, including de novo variants, compound heterozygotes, and recessive inherited variants, were generated based on Gemini (version 0.19.1). The functional assessments, including functional prediction algorithms, conservation scores, and ensemble scores, were computed using GERP +  + [19], CADD [20], SIFT [21], and Polyphen-2 [22]. PCR-Sanger sequencing was performed to validate the candidate disease-related variants detected by WES based on Applied Biosystems 3730xl DNA Analyzer (Thermo Fisher Scientific, Waltham, MA, USA). The PCR program was followed: 95 °C for 3 min; 94 °C for 30 s, 58 °C for 30 s, 72 °C for 40 s (38 cycles); 72 °C for 8 min. The sequencing results were aligned to reference sequences through CodonCode Aligner (version 6.0.2.6; CodonCode, Centerville, MA, USA).

### Bioinformation analysis

Gene ontology (GO) and Kyoto Encyclopedia of Genes and Genomes (KEGG) pathway enrichment analyses were conducted for all the candidate variants detected by WES. The online software and human genome databases, including 1000 Genomes Project Phase 3 (Han Chinese in Beijing China), Mutation Taster, Polyphen-2, ACMG, and Mendelian Clinically Applicable Pathogenicity (M-CAP) Score, were applied to identify mutation frequencies and predict the functional effects of the variants.

### Statistical analysis

Categorical variables were presented as number (percentage), and continuous variables were presented as median (interquartile range, IQR). Comparisons of categorical data were made by chi-square test. Comparisons of continuous data were made by the Wilcoxon rank-sum test. A two-side *p* < 0.05 was considered as statistically significant. All statistical analyses were performed using SPSS V26.0 for statistics and R V4.2.0 for visualization.

## Results

### Clinical characteristics of pSS-PAH patients

The demographic and clinical manifestations of the patients are shown in Table 1. The patients were mainly female (97.06%), with a median age at onset of symptoms attributable to pSS of 33.50 years (range, 29.25–40.00 years) and a median age at onset of symptoms attributable to PAH of 34.00 years (range, 30.50–41.50 years). The profiles of autoantibodies included the presence of anti-SSA in 31 (91.18%) and anti-SSB in 11 (32.25%) cases. The majority of patients were consistent with the WHO functional class II (82.35%). Seven (20.59%) patients had a family history of rheumatic diseases.

**Table 1:** Patient clinical characteristics

***N* = 34**	
**Gender, *n* (%)**	
Male	1 (2.94)
Female	33 (97.06)
**Age of pSS onset, median (IQR), years**	33.50 (29.00,38.50)
**Age of PAH onset, median (IQR), years**	34.00 (30.50,41.50)
**Disease duration of pSS at PAH onset, median (IQR), months**	1.00 (1.00,15.50)
**Clinical manifestations of pSS, *n* (%)**	
Ocular symptoms	15 (44.12)
Oral symptoms	24 (70.59)
Ocular signs	28 (82.35)
Salivary gland involvement	30 (88.24)
Histopathology	14 (41.18)
**Autoantibody positivity, *n* (%)**	
ANA	34 (100.00)
Anti-SSA	31 (91.18)
Anti-SSB	11 (32.35)
Anti-Ro52	28 (82.35)
Anti-RNP	6 (17.65)
**Organ involvement, *n* (%)**	
Hematologic	6 (17.65)
Mucocutaneous	1 (2.94)
Musculoskeletal	1 (2.94)
Gland	2 (5.88)
**SSDDI, median (IQR)**	1.00 (0.75,2.00)
**WHO cardiac function class, *n* (%)**	
I	1 (2.94)
II	28 (82.35)
III	5 (14.71)
**Family history, *n* (%)**	7 (20.59)

*Abbreviations*: *pSS* primary Sjögren’s syndrome, *PAH* Pulmonary arterial hypertension, *IQR* Interquartile range, *ANA* Antinuclear antibody, *Anti-SSA* Anti-Sjogren’s syndrome antigen A, *Anti-SSB* Anti-Sjogren’s syndrome antigen B, *Anti-RNP* Anti–ribonucleoprotein antibodies, *SSDDI* pSS disease damage index, *WHO* World Health Organization

### Identification of variants from whole exome sequencing

**Fig. 1:**
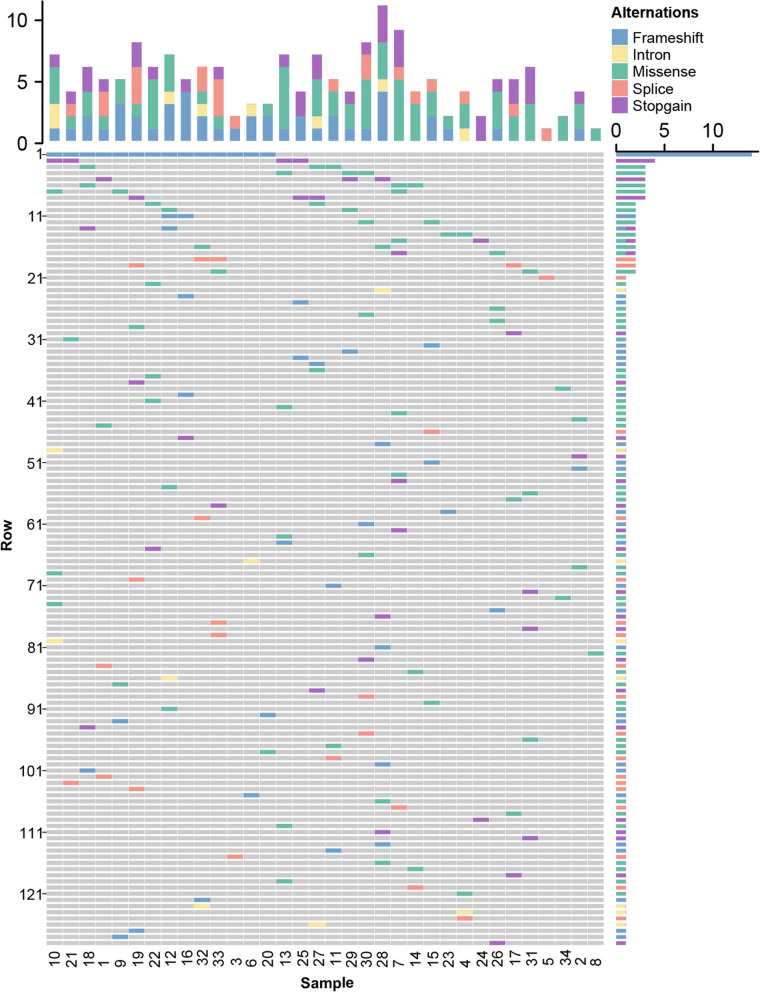
Susceptibility genes of primary Sjögren’s syndrome-associated pulmonary arterial hypertension identified by whole genome sequencing. **Gene annotation: 1-10: **BCR, FLG, CRB1, GIGYF2, ILDR1, ITK, LIPH, PRKRA, DYSF, ERCC2; **11-20: **FMN2, GJB4, LAMC3, MLH1, MUTYH, NPHP4, SERPINB7, SLC26A4, SOHLH1, TNNI3; **21-30:** ABCC6, ACADS, ACTN2, ADAMTS13, ADAMTS19, ADCY6, AGL, ALDH6A1, AQP5, ASPM; **31-40: **ATIC, ATP11B, ATP1A3, ATP7A, BAP1, BBS7, BMP4, BRWD1, BSND, CBWD2; **41-50: **CLIC6, CLPP, COL12A1, CRB2, CRYGS, CYP27A1, DDX41, DLL3, DMGDH, DNAAF1; **51-60: **DNAH8, DNAJC2, DPYS, DUOXA2, ENO3, EPB41L4A, ERCC5, EYS, FAM214A, FANCE; **61-70: **FBXL4, FIG4, FKRP, FMO3, FUT2, FZD10, GABRB3, GALK1, GCDH, GET4; **71-80: **GJB2, HEXA, HEXB, IDH1, IFT122, INPPL1, INVS, KAT2B, KIAA0586, KIF1B; **81-90: **KPNA4, KRT81, LARS2, LIPA, LIPC, MMACHC, MYO3A, MYOC, NOTCH3, NPR2; **91-100: **OPA3, PCDH15, PDE11A, PDE2A, PEX1, POLR1C, POSTN, PRPF8, RAPGEF5, RELN; **101-110: **SAMHD1, SARS2, SBDS, SCAF4, SHANK3, SLC12A3, SLC14A1, SLC22A5, SLC25A38, SLC26A8; **111-120:** SLC34A2, SLC5A5, SPAG9, SPG20, SPINK1, SPTLC2, TALDO1, TAT, TGM1, TMEM67, TPMT; **121-129: **TTN, TTN-AS1, TUBB1, UHRF1BP1L, VPS13B, VPS54, WRN, WWP1

A total of 141 pathogenic variant loci of 129 genes were identified by WES (Fig. 1, Additional file 1). In 34 patients, each patient carries 1 to 11 candidate pathologic variants. Variations of the following genes were identified in more than 1 patients: *BCR* (41.18%, *n* = 14), *FLG* (11.76%, *n* = 4), *CRB1* (8.82%, *n* = 3), *GIGYF2* (8.82%, *n* = 3), *ILDR1* (8.82%, *n* = 3), *ITK* (8.82%, *n* = 3), *LIPH* (8.82%, *n* = 3), *PRKRA* (8.82%, *n* = 3), *DYSF* (5.88%, *n* = 2), *ERCC2* (5.88%, *n* = 2), *FMN2* (5.88%, *n* = 2), *GJB4* (5.88%, *n* = 2), *LAMC3* (5.88%, *n* = 2), *MLH1* (5.88%, *n* = 2), *MUTYH* (5.88%, *n* = 2), *NPHP4* (5.88%, *n* = 2), *SERPINB7* (5.88%, *n* = 2), *SLC26A4* (5.88%, *n* = 2), *SOHLH1* (5.88%, *n* = 2), and *TNNI3* (5.88%, *n* = 2)*.* Missense, frameshift, stop-gain, splicing, and intronic were the major five types of variants found in this cohort, accounting for 38.46%, 25.44%, 18.93%, 12.43%, and 4.73% of the total mutations, respectively. Missense variation was the main variant type in *CRB1, DYSF, ERCC2*, *GIGYF2*, *GJB4*, *ITK*, *LIPH*, *MLH1*, *MUTYH*, *NPHP4*, *SERPINB7*, and *TNNI3*. Frameshift variation was the main variant type in *BCR, FMN2*, and *LAMC3*. Stop-gain variation was the main variant type in *FLG*, *ILDR1*, *LAMC3*, *MUTYH, PRKRA*, and *SERPINB7*. Splice variation was the main variant type in *SLC26A4* and *SOHLH1.*

### Function enrichment of susceptibility genes

**Fig. 2:**
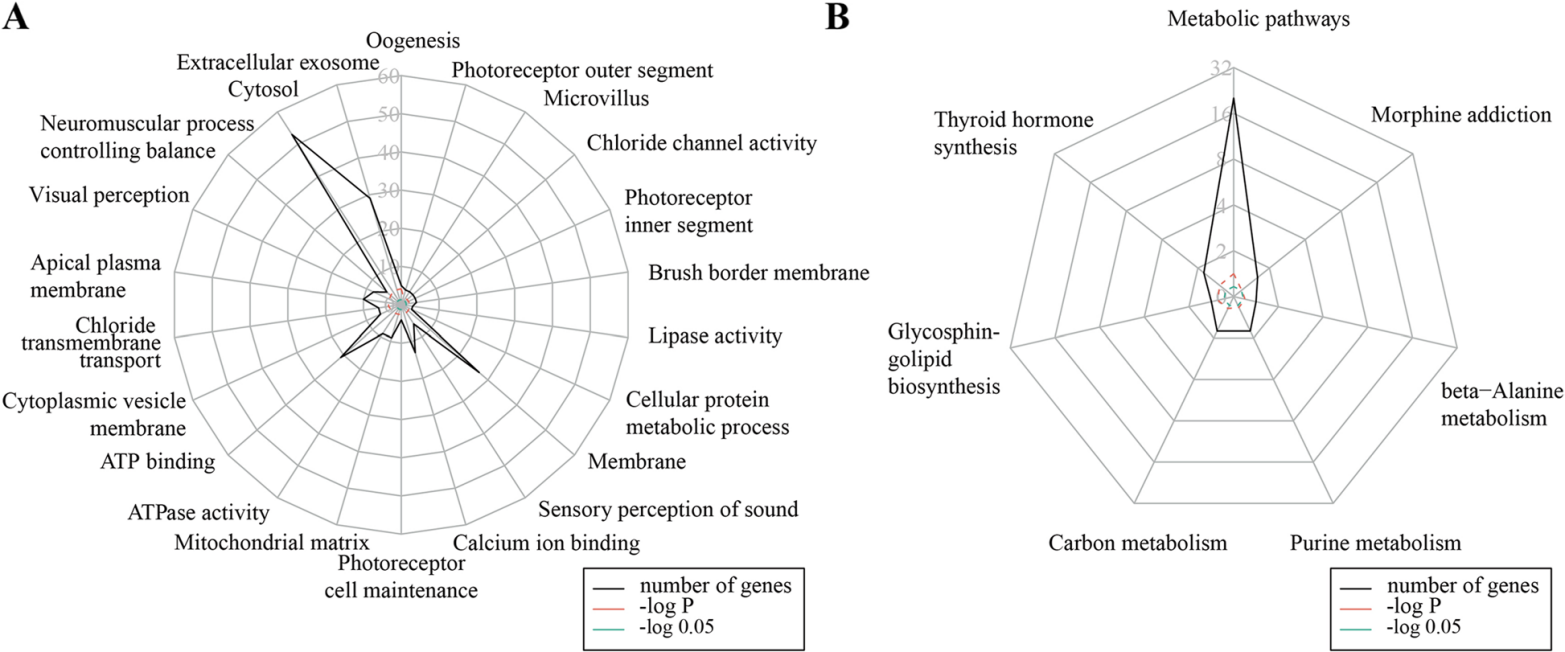
Function enrichment of susceptibility genes of primary Sjögren’s syndrome-associated pulmonary arterial hypertension. **A** Gene ontology (GO) pathway enrichment analyses; **B** Kyoto Encyclopedia of Genes and Genomes (KEGG) pathway enrichment analyses

The pathway analysis yielded 22 GO terms with a *p*-value < 0.01 and 7 KEGG terms with a *p*-value < 0.05 (Fig. 2). The GO terms with the greatest number of genes were “cytosol” (41.09%, 53 out of 129 genes), “extracellular exosome” (22.48%, 29 out of 129 genes), “membrane” (20.93%, 27 out of 129 genes), “ATP binding” (16.28%, 21 out of 129 genes), “calcium ion binding” (10.08%, 13 out of 129 genes), “apical plasma membrane” (7.75%, 10 out of 129 genes), “visual perception” (6.20%, 8 out of 129 genes). The KEGG terms with the greatest number of genes were “metabolic pathways” (20.16%, 26 out of 129 genes), “purine metabolism” (3.88%, 5 out of 129 genes), “thyroid hormone synthesis” (3.88%, 5 out of 129 genes), and “carbon metabolism” (3.88%, 5 out of 129 genes).

### Correlation between genotype and phenotype

**Fig. 3:**
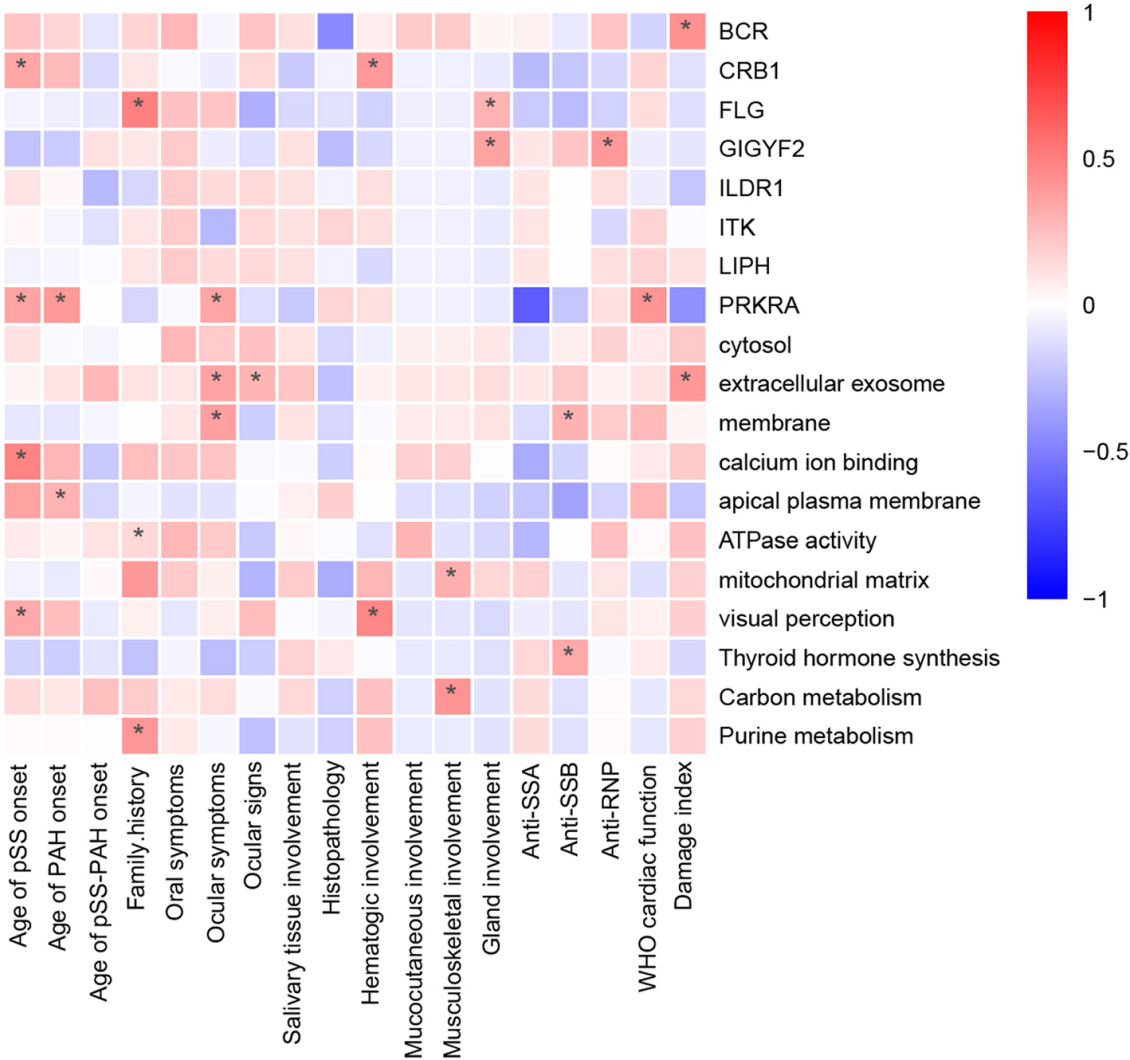
Correlation analysis of genotype and phenotype of patients with pSS-PAH. pSS, primary Sjögren’s syndrome; PAH, pulmonary arterial hypertension; disease duration, disease duration of primary Sjögren’s syndrome at pulmonary arterial hypertension onset; WHO-FC, the World Health Organization (WHO) functional class. *p < 0.05

Correlation analysis between genotypes and observed phenotypes in the patients with pSS-PAH (Fig. 3) found that patients carrying *FLG* mutations (*r* = 0.491, *p* < 0.01) and those with gene variations involved in the purine pathway (*r* = 0.405, p < 0.01) were prone to having family history of rheumatic diseases. *BCR* variations (*r* = 0.429, *p* < 0.01) and gene variations involved in the extracellular exosome pathway (*r* = 0.404, *p* < 0.01) were associated with higher SSDDI scores. Patients carrying *PRKRA* variations (*r* = 0.412, *p* < 0.01) were prone to have a higher WHO cardiac function class.

### Validation of susceptibility genes and pathogenicity prediction

**Fig. 4:**
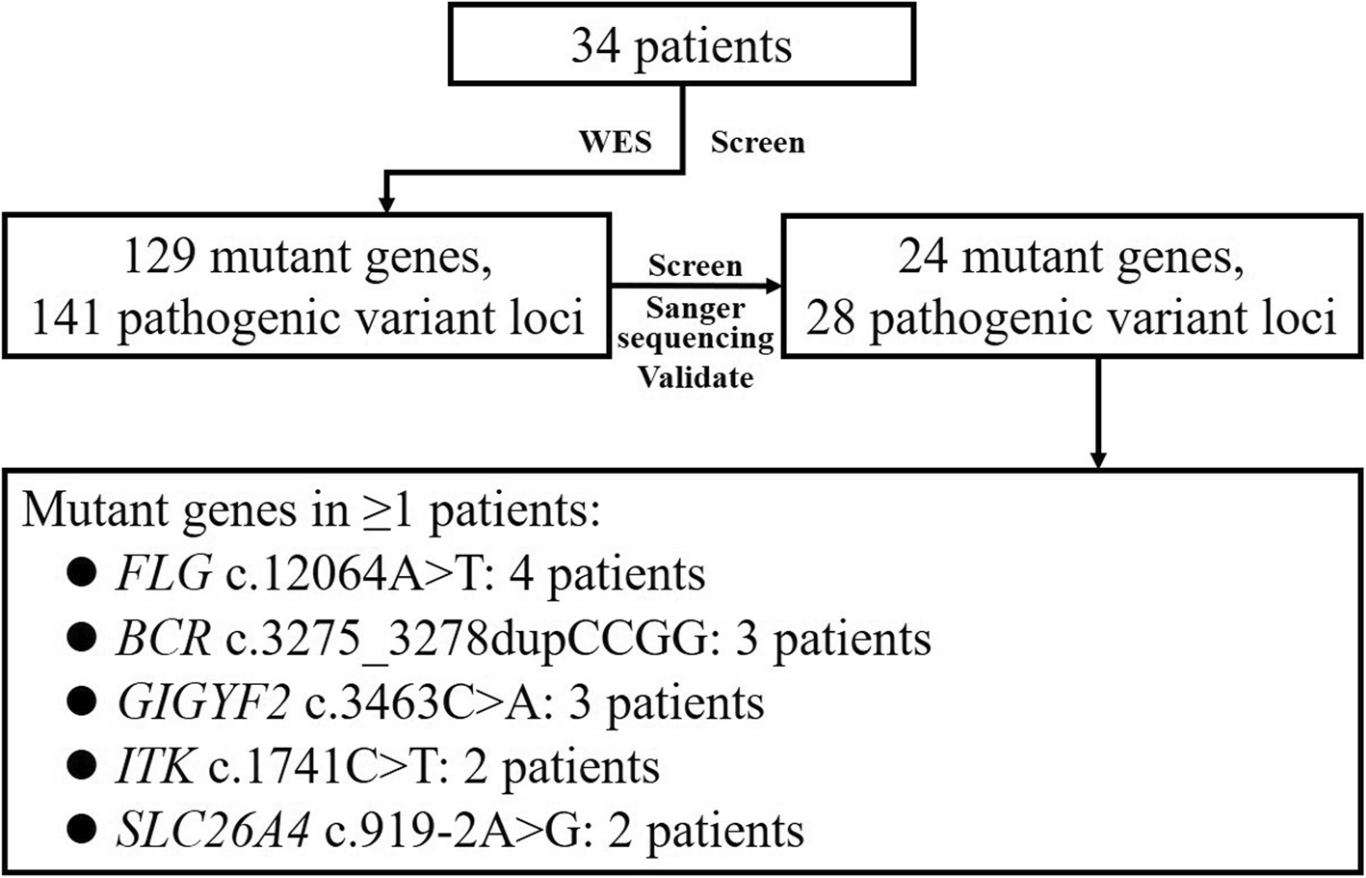
Flowchart of screening of susceptibility genes. WES, whole exome sequencing

Genes identified in more than one patient or identified in patient(s) with family history were confirmed in the Sanger sequencing. A total of 28 susceptibility variant loci from 24 genes were confirmed (Fig. 4, Additional file 2). The following pathogenic variants were identified in more than one patient: *FLG* c.12064A > T (*n* = 4), *BCR* c.3275_3278dupCCGG (*n* = 3), *GIGYF2* c.3463C > A (*n* = 3), *ITK* c.1741C > T (*n* = 2), and *SLC26A4* c.919-2A > G (*n* = 2). These variants, except for *SLC26A4*, were all located in exons and resulted in amino acid substitutions or truncation (Table 2). In addition, MutationTaster programs predicted c.12064A > T in *FLG*, c.3275_3278dupCCGG in *BCR*, c.1741C > T in *ITK*, and c.919-2A > G in *SLC26A4* were disease-causing mutations. According to the ACMG criteria, these four variants were moderate pathogenic variants.

**Table 2:** Pathogenicity prediction of variants using bioinformatics tool

Gene	Transcript	Variant position	Nucleotide change	Amino acid change	Mutation frequency	Polyphen2	MutationTaster	M-CAP	Pathogenicity (ACMG)	Evidence (ACMG)	Phenotypic effect of variant	Reference
*FLG*	NM_002016.1	Exon 3	c.12064A > T	p.Lys4022Ter	0.0049	/	Disease causing	Likely benign	Moderate pathogenicity	PM2, PP5	Ichthyosis vulgaris	[23, 24]
*BCR*	NM_004327.3	Exon 19	c.3275_3278dupCCGG	p.Val1094ArgfsTer17	/	/	Disease causing	/	Moderate pathogenicity	PM2, PP5	/	/
*GIGYF2*	NM_001103146.1	Exon 29	c.3463C > A	p.Pro1155Thr	0.0000	Probably damaging, 1.000	Polymorphism	/	Supporting benign	BP4, BP6	Early-onset Parkinson disease	[25]
*ITK*	NM_005546.3	Exon 16	c.1741C > T	p.Arg581Trp	0.0005	Probably damaging, 1.000	Disease causing	/	Moderate pathogenicity	PM2, PP5	Autoinflammatory syndrome; lymphoproliferative syndrome 1	[26, 27]
*SLC26A4*	NM_000441.1	Intron 7	c.919-2A > G	/	0.0000	/	Disease causing	Possibly pathogenic	Moderate pathogenicity	PP5	/	/

## Discussion

This is the first WES study aiming to find genetic variants associated with pSS-PAH. In the present study, we identified pathogenic variants in *FLG*, *BCR*, *ITK*, and *SLC26A4*, and one likely pathogenic variant in *GIGYF2* through WES and subsequent Sanger sequencing confirmation. Furthermore, patients with variants in *FLG* are more likely to have a family history of rheumatic diseases.

The subjects enrolled in our study were incident or prevalent pSS-PAH patients with regular medical follow-up in our center. PAH is a rare and severe complication of pSS, characterized by hypertrophy and remodeling of the right ventricle [4, 28]. With the development of genetic technology such as whole-genome and whole-exome sequencing, several key genes were identified in patients with familial PAH and IPAH, especially *BMPR2*. Further analysis from cohorts of patients with CTD-PAH, mainly with SSc-PAH, has identified additional susceptibility genes including *TBX4*, *ABCC8*, *KCNA5*, and *GDF2*/*BMP9* [9, 29]. To the best of our knowledge, the genetic features have not been reported in pSS-PAH patients worldwide. This pilot study is the first to explore genetic susceptibility of this severe complication of Sjögren’s syndrome. Our study demonstrated that several novel genes, but not susceptible genes in IPAH and other CTD-PAH, may determine the genetic susceptibility of developing pSS-PAH.

Patients with interleukin-2-inducible T-cell kinase (ITK) deficiency is prone to lymphoproliferative diseases, including Hodgkin and non-Hodgkin lymphoma, EBV lymphoproliferative disease, and hemophagocytic lymphohistiocytosis [30]. A recent study in a family with two pSS patients (sisters) identified *ITK* c.1741C > T in both probands and one unaffected sister, but not in another unaffected sister. Further bioinformatic analyses confirmed *ITK* is an immune-related gene playing a role in regulating T cell differentiation and development and T-cell receptor proximal signaling [31]. Though it was elucidated that the aberrant *ITK* is associated with pulmonary inflammation through T cell regulation and oxidative-stress mechanisms [32], this gene has not been elucidated in the pathogenesis of PAH. Our study confirmed disease-causing variant, *ITK* c.1741C > T, in the exon 16 of the ITK gene occurred not only in patients with pSS, but also in patients with pSS-PAH. Additional studies are required to explore the potential role of *ITK* for pulmonary vascular involvement in pSS. In addition, *ITK* inhibitor ibrutinib may be a potential treatment for pSS-PAH [33].

Solute carrier family 26 member 4 (*SLC26A4*), which maps to chromosome 7 at q22.3, encodes a membrane protein (pendrin) responsible for the anion (especially chloride) exchange between the cytosol and extracellular space in the inner ear and thyroid gland. Moreover, its genetic and epigenetic abnormalities have been identified in cancers such as prostate cancer [34], thyroid cancer [35], and acute myoid leukemia [36]. Another study illustrated that the mutant *SLC26A4* results in the excessive accumulation of chloride in the cytoplasm and thus induces cell apoptosis by inhibiting PI3K/Akt/mTOR pathway phosphorylation [37]. PI3K/Akt/mTOR pathway has a strong link with the occurrence of PAH [38]. In the present study, we observed that a pathogenic variant of the SLC26A4 gene may be involved with the risk of developing pSS-PAH. Replication in other CTD-PAH cohorts will be important to estimate the contribution of *SLC26A4*.

We also reported disease-causing variants in the gene BCR activator of RhoGEF and GTPase (*BCR*) and the gene encoding filaggrin (*FLG*), and a probably damaging variant in the Grb10 interacting GYF protein 2 (*GIGYF2*) gene. Furthermore, it was demonstrated that variations in the gene *BCR* were significantly associated with organ damage accrual in patients with pSS-PAH. We also detected a significant phenotype-genotype correlation between the gene *FLG* and the family history of rheumatic and musculoskeletal diseases among these pSS-PAH patients. The *BCR* gene, located on chromosome 22, is most known as the breakpoint for chromosomes 22 and 9 reciprocal translocation, which produces the Philadelphia chromosome and is common in patients with chronic myelogenous leukemia [39]. Although the fusion gene has been extensively studied in the pathogenesis of leukemia, the function of *BCR* and whether it is a potential trigger to other tumors and diseases are not clear yet. *FLG* variants are the most replicated and strongest genetic risk factors for eczema and eczema-associated asthma [40]. Furthermore, *FLG* variants participate in susceptibility to psoriasis, as well as other autoimmune and skin disorders [41, 42]. *GIGYF2* variants are of interest for their important role in familial Parkinson’s disease [25, 43]. In addition, GIGYF2 protein was identified as an adapter protein that binds activated IGF-I and insulin receptors [44]. Thus, our study suggests for the first time the roles of *BCR*, *FLG*, and *GIGYF2* in the pathogenesis of pSS-PAH.

The study on susceptibility genes of multifactorial diseases, like pSS-PAH, remains challenging. Although our sample size was relatively small and we lack the data of the control group, this is the first WES study which clarifies the genotype–phenotype correlations in patients with pSS-PAH. Further studies are necessary to recruit healthy controls, pSS patients without PAH and IPAH patients, and large cohort of patients with pSS-PAH to conduct site-based association analysis for common variants and gene-based burden analysis for rare variants. Furthermore, more experiments are needed to illuminate the expression and related functions of these candidate genes.

## Conclusion

Using WES on rare diseases cohort, our work firstly identified novel susceptibility genes associated with pSS-PAH. These variants in *FLG*, *BCR*, *GIGYF2*, *ITK*, and *SLC26A4* may serve as potential biomarkers in Chinese pSS-PAH patients.

### Supplementary Information

**Additional file 1: Supplementary Table 1. **Susceptibility genes of primary Sjögren’s syndrome-associated pulmonary arterial hypertension identified by whole genome sequencing.

**Additional file 2: Supplementary Figure 1. **Variants found in *FLG*, *BCR*, *GIGYF2*, *ITK*, and *SLC26A4* verified by Sanger sequencing.

## Data Availability

The original contributions presented in the study are included in the article. Further inquiries can be directed to the corresponding author.

## Abbreviations

PAH: Pulmonary arterial hypertension
pSS: Primary Sjögren’s syndrome
WES: Whole-exome sequencing
GO: Gene ontology
KEGG: Kyoto Encyclopedia of Genes and Genomes
PCR: Polymerase chain reaction
CTD: Connective tissue disease
IPAH: Idiopathic PAH
HPAH: Heritable PAH
SLE: Systemic lupus erythematosus
SSc: Systemic sclerosis
BMPR2: Bone morphogenic protein receptor type 2
BMP9: Bone morphogenetic protein 9
PTGIS: Prostacyclin synthase
PUMCH: Peking Union Medical College Hospital
ACR: American College of Rheumatology
EULAR: European League Against Rheumatism
RHC: Right heart catheterization
mPAP: Mean pulmonary arterial pressure
PAWP: Pulmonary artery wedge pressure
V/Q: Ventilation perfusion scintigraphy
CTPA: Computed tomographic pulmonary angiography
SSDDI: PSS disease damage index
WHO: World Health Organization
ITK: Interleukin-2-inducible T-cell kinase
SLC26A4: Solute carrier family 26 member 4
FLG: Filaggrin
GIGYF2: Grb10 interacting GYF protein 2
