# Supplementary material for: Identification of potential susceptibility genes in patients with primary Sjögren’s syndrome-associated pulmonary arterial hypertension through whole exome sequencing
Source: Arthritis Res Ther. 2023 Sep 20;25:175. doi: 10.1186/s13075-023-03171-y (PMC10510152; doi:10.1186/s13075-023-03171-y)
Supplement: Supplementary file 1 — Additional file 1: Supplementary Table 1. Susceptibility genes of primary Sjögren’s syndrome-associated pulmonary arterial hypertension identified by whole genome sequencing. [file 13075_2023_3171_MOESM1_ESM.docx]

**Identification of potential susceptibility genes in patients with primary Sjögren’s syndrome associated pulmonary arterial hypertension through whole exome sequencing**

**Supplementary Table 1.** Susceptibility genes of primary Sjögren’s syndrome-associated pulmonary arterial hypertension identified by whole genome sequencing.

| **Gene** | **Variant position** | **Nucleotide change** | **Alternation** | **Case** |
| --- | --- | --- | --- | --- |
| *ABCC6* | Intron 30 | c.4404-1G>A(-) | Splicing | 1 |
| *ACADS* | Exon 10 | c.1130C>T(p.Pro377Leu) | Missense | 1 |
| *ACTN2* | Intron 8 | c.784-524delA | Intronic | 1 |
| *ADAMTS13* | Exon 12 | c.1335delC (p.Phe445LeufsTer52);  c.1242delC (p.Phe414LeufsTer52);  c.1335delC (p.Phe445LeufsTer52);  n.993-2512delC | Frameshift | 1 |
| *ADAMTS19* | Exon 12 | c.1913_1914dupAG (p.Trp639SerfsTer26) | Frameshift | 1 |
| *ADCY6* | Exon 18 | c.2887C>T (p.Arg963Trp) | Missense | 1 |
| *AGL* | Exon 25 | c.3347G>A (p.Arg1116His) | Missense | 1 |
| *ALDH6A1* | Exon 11 | c.1478G>T (p.Gly493Val) | Missense | 1 |
| *AQP5* | Exon 1 | c.190A>G (p.Ser64Gly) | Missense | 1 |
| *ASPM* | Exon 18 | c.8314C>T (p.Gln2772Ter) | Stop_gain | 1 |
| *ATIC* | Exon 16 | c.1721C>A (p.Ala574Asp) | Missense | 1 |
| *ATP11B* | Exon 3 | c.173delA (p.Asn58IlefsTer23) | Frameshift | 1 |
| *ATP1A3* | Exon 20 | c.2708delT (p.Val903GlyfsTer12) | Frameshift | 1 |
| *ATP7A* | Exon 23 | c.4217dupT (p.Leu1407ProfsTer2) | Frameshift | 1 |
| *BAP1* | Exon 5 | c.362delG (p.Gly121ValfsTer66) | Frameshift | 1 |
| *BBS7* | Exon 4 | c.187G>A (p.Gly63Arg) | Missense | 1 |
| *BCR* | Exon 19 | c.3275_3278dupCCGG(p.Val1094ArgfsTer17) | Frameshift | 14 |
| *BMP4* | Exon 4 | c.485G>A(p.Arg162Gln) | Missense | 1 |
| *BRWD1* | Exon 30 | c.3470G>A (p.Trp1157Ter) | Stop_gain | 1 |
| *BSND* | Exon 4 | c.893G>A (p.Gly298Glu) | Missense | 1 |
| *CBWD2* | Exon 10 | c.755_756delCT (p.Ser252TrpfsTer19) | Frameshift | 1 |
| *CLIC6* | Exon 4/5 | c.1651G>A (p.Ala551Thr) | Missense | 1 |
| *CLPP* | Exon 4 | c.425C>T(p.Pro142Leu) | Missense | 1 |
| *COL12A1* | Exon 47 | c.7438G>A(p.Val2480Met) | Missense | 1 |
| *CRB1* | Exon 7 | c.3061G>A(p.Val1021Met) | Missense | 1 |
|  | Exon 3 | c.664G>A (p.Glu222Lys) | Missense | 1 |
|  | Exon 9 | c.3397G>A (p.Val1133Met) | Missense | 1 |
| *CRB2* | Exon 11 | c.3448T>C(p.Cys1150Arg) | Missense | 1 |
| *CRYGS* | Exon 3 | c.297T>G(p.Phe99Leu) | Missense | 1 |
| *CYP27A1* | Intron 7 | c.1263+1G>A | Splicing | 1 |
| *DDX41* | Exon 8 and 4872 bp to transcript | c.797C>A (p.Ser266Ter) | Stop_gain | 1 |
| *DLL3* | Exon 6 | c.920delT (p.Leu307ArgfsTer5) | Frameshift | 1 |
| *DMGDH* | 206 bp to transcript | - | Upstream_gene | 1 |
| *DNAAF1* | Exon 7 | c.994G>T(p.Glu332Ter) | Stop_gain | 1 |
| *DNAH8* | Exon 92 | c.13731delG (p.Met4577IlefsTer5) | Frameshift | 1 |
| *DNAJC2* | Exon 9 | c.858delA(p.Glu287LysfsTer31) | Frameshift | 1 |
| *DPYS* | Exon 4 | c.637G>C(p.Gly213Arg) | Missense | 1 |
| *DUOXA2* | 778 bp to transcript | - | Stop_gain | 1 |
| *DYSF* | Exon 43 | c.4742G>A(p.Arg1581His) | Missense | 1 |
|  | Exon 30 | c.3289C>T (p.Arg1097Cys) | Missense | 1 |
| *ENO3* | Exon 2 | c.46G>C(p.Gly16Arg) | Missense | 1 |
| *EPB41L4A* | Exon 2 | c.111A>T (p.Lys37Asn) | Missense | 1 |
| *ERCC2* | Exon 8 | c.691G>A(p.Val231Met) | Missense | 1 |
|  | Exon 21 | c.1922G>A (p.Arg641Gln) | Missense | 1 |
| *ERCC5* | Exon 13 and 21 | c.2831C>G (p.Ser944Trp) | Missense | 1 |
| *EYS* | Exon 43 | c.8545C>T (p.Arg2849Ter) | Stop_gain | 1 |
| *FAM214A* | Exon 8 | c.2539delA (p.Ser847ValfsTer6) | Frameshift | 1 |
| *FANCE* | Intron 2 | c.855+2T>C | Splicing | 1 |
| *FBXL4* | Exon 5 | c.576dupA (p.Pro193ThrfsTer4) | Frameshift | 1 |
| *FIG4* | Exon 19 | c.2158G>T(p.Glu720Ter) | Stop_gain | 1 |
| *FKRP* | Exon 4 | c.328C>T(p.Arg110Trp) | Missense | 1 |
| *FLG* | Exon 3 | c.12064A>T(p.Lys4022Ter) | Stop_gain | 4 |
| *FMN2* | Exon 5 | c.2857delA(p.Ile953TyrfsTer322) | Frameshift | 2 |
|  | Exon 5 | c.2852_2853insA(p.Ala952GlyfsTer301) | Frameshift | 2 |
| *FMO3* | Exon 5 | c.591_592delTG(p.Cys197Ter) | Frameshift | 1 |
| *FUT2* | Exon 2 | c.604C>T(p.Arg202Ter) | Stop_gain | 1 |
| *FZD10* | Exon 1 | c.1341C>G (p.Phe447Leu) | Missense | 1 |
| *GABRB3* | Intron 3 | c.241-7465G>A(-) | Intron | 1 |
| *GALK1* | Exon 8 | c.1159G>A(p.Ala387Thr) | Missense | 1 |
| *GCDH* | Exon 11 | c.1240G>A(p.Glu414Lys) | Missense | 1 |
| *GET4* | Intron 2 | c.234+1G>C | Splicing | 1 |
| *GIGYF2* | Exon 2 | c.3463C>A(p.Pro1155Thr) | Missense | 3 |
| *GJB2* | Exon 2 | c.299_300delAT(p.His100ArgfsTer14) | Frameshift | 1 |
| *GJB4* | Exon 2 | c.292C>T (p.Arg98Cys) | Missense | 1 |
|  | Exon 2 | c.271G>A (p.Val91Met) | Missense | 1 |
| *HEXA* | Exon 3 | c.409C>T (p.Arg137Ter) | Stop_gain | 1 |
| *HEXB* | Exon 14 | c.1615C>T (p.Arg539Cys) | Missense | 1 |
| *IDH1* | Exon 6 | c.623A>G(p.Tyr208Cys) | Missense | 1 |
| *IFT122* | Exon 28 | c.3451dupG (p.Glu1151GlyfsTer5) | Frameshift | 1 |
| *ILDR1* | Exon 6 | c.772C>T (p.Gln258Ter) | Stop_gain | 3 |
| *INPPL1* | Exon 10 | c.1111C>T (p.Gln371Ter) | Stop_gain | 1 |
| *INVS* | Intron 7 | c.906+1G>A | Splice_donor | 1 |
| *ITK* | Exon 16 | c.1741C>T(p.Arg581Trp) | Missense | 3 |
| *KAT2B* | Exon 4 | c.631G>T (p.Glu211Ter) | Stop_gain | 1 |
| *KIAA0586* | Intron 11 | c.1584-2A>G | Splicing | 1 |
| *KIF1B* | Intron 20 | c.1977+6535C>T(-) | Intron | 1 |
| *KPNA4* | Exon 15 | c.1344delC (p.Ile449Ter) | Frameshift | 1 |
| *KRT81* | Exon 1 | c.241G>T(p.Val81Leu) | Missense | 1 |
| *LAMC3* | Exon 4 | c.937_944delTGGGCCCG(p.Trp313GlyfsTer24) | Frameshift | 1 |
|  | Exon 4 | c.955G>T (p.Glu319Ter) | Stop_gain | 1 |
| *LARS2* | Exon 18 | c.2080C>T (p.Arg694Ter) | Stop_gain | 1 |
| *LIPA* | Intron 1 | c.-1-1G>C(-) | Splicing | 1 |
| *LIPC* | Exon 2 | c.212C>T(p.Thr71Met) | Missense | 1 |
| *LIPH* | Exon 6 | c.742C>A(p.His248Asn) | Missense | 2 |
|  | Exon 2 | c.329G>T(p.Arg110Leu) | Missense | 1 |
| *MLH1* | Exon 8 | c.649C>T(p.Arg217Cys) | Missense | 2 |
| MMACHC | 3544 bp to transcript | - | Downstream_gene | 1 |
| *MUTYH* | Exon 10 | c.815G>A(p.Gly272Glu) | Missense | 1 |
|  | Exon 3 | c.289C>T (p.Arg97Ter) | Stop_gain | 1 |
| *MYO3A* | Exon 6 | c.426T>G(p.His142Gln) | Missense | 1 |
| *MYOC* | Exon 1 | c.136C>T (p.Arg46Ter) | Stop_gain | 1 |
| *NOTCH3* | Intron 26 | c.4891+1G>C | Splicing | 1 |
| *NPHP4* | Exon 17 | c.2198G>A (p.Gly733Asp) | Missense | 1 |
|  | Exon 22 | c.3160C>T (p.Arg1054Cys) | Missense | 1 |
| *NPR2* | Exon 15 | c.2359C>T (p.Arg787Trp) | Missense | 1 |
| *OPA3* | Exon 1 | c.123C>G(p.Ile41Met) | Missense | 1 |
| *PCDH15* | Exon 34\35\36 | c.4867_4870dupGACA (p.Asn1624ArgfsTer17)  c.4852_4855dupGACA (p.Asn1619ArgfsTer17) | Frameshift | 1 |
| *PDE11A* | Exon 2 | c.20_21delGA(p.Arg7ThrfsTer30) | Frameshift | 1 |
| *PDE2A* | Intron 2 | c.144+11168C>T | Stop_gain | 1 |
| *PEX1* | Intron 3 | c.357+1G>T | Splicing | 1 |
| *POLR1C* | Exon 8 | c.917A>G (p.Tyr306Cys) | Missense | 1 |
| *POSTN* | Exon 1 | c.1733A>T(p.Glu578Val) | Missense | 1 |
| *PRKRA* |  | c.610-1_610insGAATGCTGCTGAGAAATTTCTTGCCAAATTTAGTAATATTTCTCCAGAGAACCACATTTCTTTA (p.Thr204GlufsTer4) | Stop_gain | 3 |
| *PRPF8* | Exon 23 | c.3451C>T (p.Arg1151Trp) | Missense | 1 |
| *RAPGEF5* | Intron 17 | c.1328-1G>T(-) | Splicing | 1 |
| *RELN* | Exon 15 | c.1766_1767insTTCAATATCT (p.Leu591IlefsTer20) | Frameshift | 1 |
| *SAMHD1* | Exon 3 | c.286dupG (p.Glu96GlyfsTer7) | Frameshift | 1 |
| *SARS2* | Intron 16 | c.1420-2A>C(-) | Splicing | 1 |
| *SBDS* | Intron 2 | c.258+2T>C | Splicing | 1 |
| *SCAF4* | Intron 2 | c.115-2A>G | Splicing | 1 |
| *SERPINB7* | Exon 8 | c.796C>T(p.Arg266Ter) | Stop_gain | 1 |
|  | Exon 6 | c.455G>T (p.Gly152Val) | Missense | 1 |
| *SHANK3* | Exon 21 | c.2415_2416delCA(p.Arg806ThrfsTer) | Frameshift | 1 |
| *SLC12A3* | Exon 14 | c.1732G>A (p.Val578Met) | Missense | 1 |
| *SLC14A1* | Intron 5 | c.510-1G>A(-) | Splicing | 1 |
| *SLC22A5* | Exon7 | c.1195C>T (p.Arg399Trp) | Missense | 1 |
| *SLC25A38* | Exon 3 | c.212T>A (p.Leu71Ter) | Stop_gain | 1 |
| *SLC26A4* | Intron 7 | c.919-2A>G | Splicing | 2 |
| *SLC26A8* | Exon 9 | c.1087T>C(p.Ser363Pro) | Missense | 1 |
| *SLC34A2* | Exon 8 | c.910A>T (p.Lys304Ter) | Stop_gain | 1 |
| *SLC5A5* | Exon 9 | c.1162A>T (p.Lys388Ter) | Stop_gain | 1 |
| *SOHLH1* | Intron 3 and 4557 bp to transcript | c.346-1G>A | Splicing | 1 |
|  |  | NM_001012415.2:c.346-1G>A;  NM_001101677.1:c.346-1G>A | Splicing | 1 |
| *SPAG9* | Exon 3 | c.453delA (p.Glu151AspfsTer2) | Frameshift | 1 |
| *SPG20* | Exon 2 | c.364_365delAT(p.Met122ValfsTer2) | Frameshift | 1 |
| *SPINK1* | Intron 3 | c.194+2T>C(-) | Splicing | 1 |
| *SPTLC2* | Exon 8 | c.1067G>C (p.Gly356Ala) | Missense | 1 |
| *TALDO1* | Exon 7 | c.931G>A(p.Gly311Arg) | Missense | 1 |
| *TAT* | Exon 11 | c.1141G>T (p.Glu381Ter) | Stop_gain | 1 |
| *TGM1* | Exon 3 | c.463C>T(p.Arg155Trp) | Missense | 1 |
| *TMEM67* | Intron 1 | c.224-2delA(-) | Splicing | 1 |
| *TNNI3* | Exon 5 | c.235C>T (p.Arg79Cys) | Missense | 2 |
| *TPMT* | Exon 4 | c.238G>C(p.Ala80Pro) | Missense | 1 |
| *TTN* | Exon 287 | c.55533delC (p.Gly18513AlafsTer27) | Frameshift | 1 |
| *TTN-AS1* | Intron 16 | n.3917+797delG | Intronic | 1 |
| TUBB1 | 4766 bp to transcript | - | Downstream_gene | 1 |
| *UHRF1BP1L* | Intron 9 | c.1105+1G>T(-) | Splicing | 1 |
| *VPS13B* | Intron 27 | c.4158-634G>A | Intronic | 1 |
| *VPS54* | Exon 15 | c.2121dupA (p.Ser720IlefsTer11) | Frameshift | 1 |
| *WRN* | Exon 18 | c.2017dupA(p.Ile673AsnfsTer3) | Frameshift | 1 |
| *WWP1* | Exon 19 | c.2089G>T (p.Glu697Ter) | Stop_gain | 1 |
