# Supplementary material for: Identification of potential susceptibility genes in patients with primary Sjögren’s syndrome-associated pulmonary arterial hypertension through whole exome sequencing
Source: Arthritis Res Ther. 2023 Sep 20;25:175. doi: 10.1186/s13075-023-03171-y (PMC10510152; doi:10.1186/s13075-023-03171-y)
Supplement: Supplementary file 2 — Additional file 2: Supplementary Figure 1. Variants found in FLG, BCR, GIGYF2, ITK, and SLC26A4 verified by Sanger sequencing. [file 13075_2023_3171_MOESM2_ESM.docx]

**Identification of potential susceptibility genes in patients with primary Sjögren’s syndrome associated pulmonary arterial hypertension through whole exome sequencing**


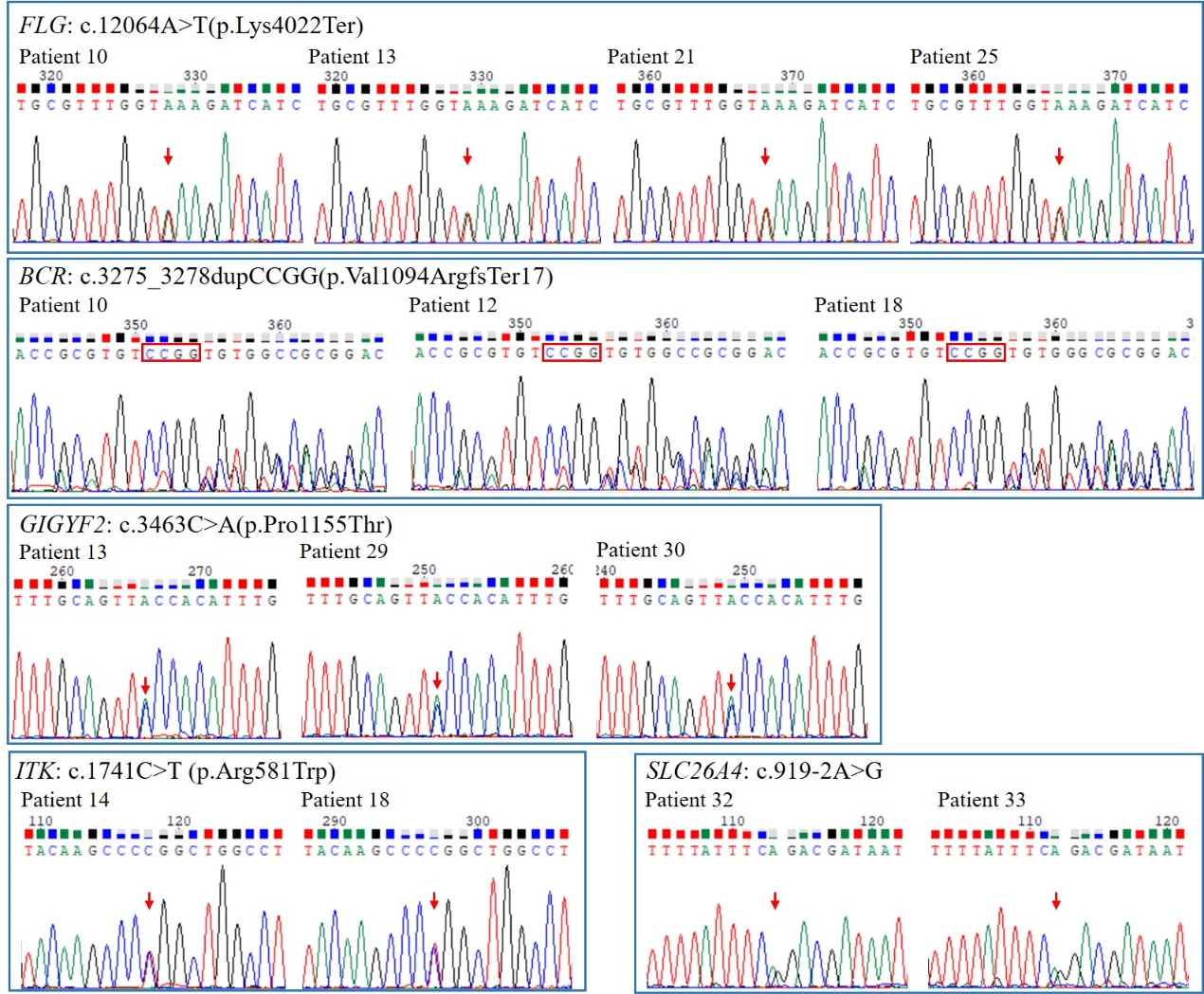


**Supplementary Figure 1.** Variants found in *FLG*, *BCR*, *GIGYF2*, *ITK*, and *SLC26A4* verified by Sanger sequencing.
